# Discussion in Regard to the Use of Digitalis, Chloral and Bromide of Potassium

**Published:** 1874-11

**Authors:** 


### Discussion in Regard to the use of Digitalis, Chloral and Bromide of Potassium.

SOCIETY OF MEDICINE, of Paris.

M. DUROZIEZ.—I desire to communicate to the society some facts in a case where the powder of digitalis was given in a dose of fifteen centigrammes in ptisan, and at the end of a very short time the patient succumbed. Ought one to attribute this rapid termination of the disease to the medicine, or to the fatty condition of the heart revealed by the autopsy? I am inclined to believe that it was the medicine which precipitated the fatal issue. There were signs, prior to death, of the toxic action of digitalis.

M. PETER.—I ask M. Duroziez by what signs he recognizes a fatty condition of the heart? For my part I find the diagnosis very difficult. Indeed a fatty condition of the heart has no signs sufficiently evident to permit the observer to establish a certain diagnosis, so that one can only arrive at a recognition of a fatty degeneration by an ensemble of probabilities, viz: feebleness of the pulse, fatty degeneration of the arteries, arcus senilis, evident chronic alcoholism.

There is the danger in digitalis, and I give it very little, and almost against my will; I do not trust it; I do not trust to it; I fear it. I employ it only as a regulator of the movements of the heart, when these latter are very irregular and tumultuous.

There is a tendency in the medicine of our day, a deplorable tendency, of establishing cut and dried equations systematically drawn between the disease and the medicine: syphilis, mercury; disease of the heart, digitalis. This is no longer science, it is a ready-made, stereotyped, conventional system; it is the triumph of mere routine.

It is not right to administer digitalis during a long time, no matter under what form it is prescribed. One can, and one ought to suspend its use, for it is well known, and can not be too much emphasized, that digitalis is an unsafe, a dangerous drug.

Now M. Duroziez has made a very interesting and useful work, and I should like to ask him to put it into form, to divide his numerous observations under different heads; that is, to bring together in the same chapter all those cases in which, digitalis having been given, troubles in the digestive function followed; in another to bring together all cases of delirium, hallucination, in a word, of of encephalic trouble; and, moreover, at once, in order that this conscientous and interesting work may not lose its force by being published at long intervals, and pass, as it were, unnoticed by the readers of the Gazette des Hôpitaux.

It is of the greatest importance in a therapeutic point of view for country practitioners to know well the dangers of the drug, and that in aresting diseases of the heart the fear of digitalis is the beginning of wisdom.

I agree with M. Duroziez on this subject.

M. Duroziez.—I ask nothing better; I began this work without any partisan spirit, in good faith, without knowing what my conclusions would be, and while progressing with my work I became appalled at the revelations my observations made of the toxic effects of digitalis.

M. Delasianve.—I share the opinions of MM. Duroziez and Peter, and I would say as much of chloral and bromide of potassium, which are used helter skelter without discretion; if a sick man is in pain, chloral; if a patient present sympthms of any nervous condition whatever, bromide of potassium; and I am not at all convinced of the innocuousness of these substances, I believe that their influence may make itself felt for a long time, and is not without danger.

M. Forget.—When one practices medicine on symptoms alone, one practices bad science; the present moment alone occupies the attention, and the remote effect of the drug is lost sight of.

What do I do when I give chloride or bromide of potassium? How do they act on the economy? as toxic agents, as a veritable poisoning; and I am not yet convinced that they are not dangerous; at all events, there are two sorts of action; the one primary, the other secondary.

I have seen chloral dangerous for nervous women, and I have often seen them worse after the administration of the drug.

Time for experimentation is not given; the medicine in vogue is administered; it is the fashion which is followed, and not the rational study of the drug. It is not scientific experimentation, it is innovation in therapeutics, and nothing more. More prudence must be exercised, more reason, more patience, and the work of M. Duroziez will have the good fortune to make known to all the danger of the employment of digitalis.—Gaz. des Hôp., Oct. 1874. —*The Clinic.*

